# Predicting dementia from spontaneous speech using large language models

**DOI:** 10.1371/journal.pdig.0000168

**Published:** 2022-12-22

**Authors:** Felix Agbavor, Hualou Liang

**Affiliations:** School of Biomedical Engineering, Science and Health Systems, Drexel University, Philadelphia, United States of America; Massachusetts General Hospital, UNITED STATES

## Abstract

Language impairment is an important biomarker of neurodegenerative disorders such as Alzheimer’s disease (AD). Artificial intelligence (AI), particularly natural language processing (NLP), has recently been increasingly used for early prediction of AD through speech. Yet, relatively few studies exist on using large language models, especially GPT-3, to aid in the early diagnosis of dementia. In this work, we show for the first time that GPT-3 can be utilized to predict dementia from spontaneous speech. Specifically, we leverage the vast semantic knowledge encoded in the GPT-3 model to generate text embedding, a vector representation of the transcribed text from speech, that captures the semantic meaning of the input. We demonstrate that the text embedding can be reliably used to (1) distinguish individuals with AD from healthy controls, and (2) infer the subject’s cognitive testing score, both solely based on speech data. We further show that text embedding considerably outperforms the conventional acoustic feature-based approach and even performs competitively with prevailing fine-tuned models. Together, our results suggest that GPT-3 based text embedding is a viable approach for AD assessment directly from speech and has the potential to improve early diagnosis of dementia.

## Author summary

Alzheimer’s disease is a currently incurable brain disorder. Speech, a quintessentially human ability, has emerged as an important biomarker of neurodegenerative disorders like AD. Can AI-driven speech analysis help identify AD? We show in this study that GPT-3, a specific language model produced by OpenAI, could be a step towards early prediction of AD through speech. Specifically, we demonstrate that text embedding, powered by GPT-3, can be reliably used to (1) distinguish individuals with AD from healthy controls, and (2) infer the subject’s cognitive testing score, both solely based on speech data. We further show that text embedding considerably outperforms the conventional feature-based approach and even performs competitively with the mainstream use of fine-tuned models. Our results suggest that there is a huge potential to develop and translate a fully deployable AI-driven tools for early diagnosis of dementia and direct tailored interventions to individual needs, thereby improving quality of life for individuals with dementia.

## Introduction

Alzheimer’s disease (AD) is a neurodegenerative disease that involves progressive cognitive declines, including speech and language impairments. It is the most common etiology of dementia, affecting 60–80% cases [1]. Given its prevalence and still no cure available for AD treatment [2], there is an urgent need for the early diagnosis of dementia, which would yield clear benefits in improving quality of life for individuals with dementia.

Current diagnoses for AD are still primarily made through clinical assessments such as brain imaging or cognitive tests e.g., Mini-Mental State Examination (MMSE) [3] for evaluating the progression of AD [4,5]. However, they are often expensive and involve lengthy medical evaluations. Previous studies have shown that spontaneous speech contains valuable clinical information in AD [6]. The use of speech as a biomarker provides quick, cheap, accurate and non-invasive diagnosis of AD and clinical screening. Previous works on speech analysis are mainly based on the feature-based approach using acoustic features extracted from the speech audio and the linguistic features derived from the written texts or speech transcripts through NLP techniques [7]. Both the linguistic and acoustic features, sometimes along with other speech characteristics, have been extensively used for dementia classification based on speech data [8–17]. This feature-based approach, however, relies heavily upon domain specific knowledge and hand-crafted transformations. As a result, it often fails to extract more abstract, high-level representations [18,19], hence is hard to generalize to other progression stages and disease types, which may correspond to different linguistic features. AI-enabled speech and language analysis has emerged as a promising approach for early screening of Alzheimer’s dementia [9,10,15,20–22].

Large language models (LLMs), which have demonstrated impressive performance on many NLP tasks, provide powerful universal language understanding and generation [23–25]. GPT- 3, or Generative Pre-trained Transformer 3, one of the largest existing language models produced by OpenAI [26], has been shown to be particularly effective in (1) *zero-shot learning* (i.e., zero-data learning), where the language model is adapted to downstream tasks, such as translation, text summarization, question-answering and dialogue systems, without the need for additional, task-specific data [26], and (2) encoding a wealth of semantic knowledge about the world and producing a learned representation (*embedding*), typically a fixed-size vector, that lends itself well to discriminative tasks [27]. The text embeddings entail meaningful vector representations that can uncover additional patterns and characteristics, as captured in the semantic meaning of the input, that might not be evident even to trained experts. It has been extremely successful to learn text embeddings in NLP [23,24,28–30]. However, so far there is no study on the use of GPT-3 for AD detection.

**Fig 1:**
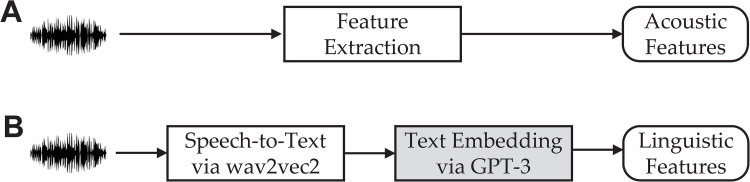
Schematic showing two different feature representations that are derived from speech. A. The acoustic features are engineered to capture the acoustic characteristics of speech and therefore the pathological speech behavior. B. The linguistic features, represented as text embeddings, are derived from the transcribed text. Central to our proposed approach is the GPT-3 based text embeddings (shaded), which entail meaningful vector representations that can capture lexical, syntactic, and semantic properties for dementia classification.

In this work, we study the extent to which text embeddings generated by GPT-3 are utilized to predict the dementia. We use the data from the ADReSSo (Alzheimer’s Dementia Recognition through Spontaneous Speech *only*) Challenge [21], a shared task for the systematic comparison of approaches to the detection of cognitive impairment and decline based on spontaneous speech. With this dataset, we perform two tasks: an AD classification task for distinguishing individuals with AD from healthy controls, and an MMSE score regression task to infer the cognitive test score of the subject, both solely based on the demographically matched spontaneous speech data. We show that the text embedding can be reliably used for detection of Alzheimer’s dementia and inference of the cognitive testing score. We further show that text embedding (Fig 1B) considerably outperforms the conventional acoustic feature-based approach (Fig 1A) and is even competitive with fine-tuned models. Taken together, our results demonstrate that text embedding, derived from GPT-3 model, is a viable approach for the assessment of AD status with great promise in assisting with early diagnosis of dementia.

## Results

We report the results from two tasks which include AD vs non-AD classification and AD severity prediction using a subject’s MMSE score. For the classification task, either the acoustic features or GPT-3 embeddings (Ada and Babbage) or both are fed into a machine-learning model such as support vector classifier (SVC), logistic regression (LR) or random forest (RF). As a comparison, we further perform finetuning on the GPT-3 model to see if there is any advantage over the GPT-3 embedding.

For the AD severity prediction, we perform the regression analysis based on both the acoustic features and GPT-3 embeddings to estimate a subject’s MMSE score using three regression models, i.e., support vector regressor (SVR), ridge regression (Ridge) and random forest regressor (RFR).

### AD vs Non-AD classification

In this section we present the AD classification results between AD and non-AD (or healthy control) subjects based on different features: our proposed GPT-3 based text embeddings, the acoustic features, and their combination. We also benchmark the GPT-3 based text embeddings against the mainstream fine-tuning approach. We show that the GPT-3 based text embeddings considerably outperform both the acoustic feature-based approach and the fine-tuned model.

### Using acoustic features

The classification performance in terms of accuracy, precision, recall and F1 score for all the models with the acoustic features is shown in Table 1 for both the 10-fold cross-validation (CV) and evaluation on the held-out test set not used in any way during model development. From Table 1, we can see that for the evaluation on the test set, RF performs the best among the three models in all the metrics used. For the 10-fold CV, RF also has the highest recall and F1 score among all the models, although the SVC performs better than other two models in both accuracy and precision.

**Table 1:** Model performance obtained by the 10-fold CV (top) where the mean (standard deviation) are reported, and evaluated on test set (bottom) for AD classification using acoustic features. Bold indicates the best overall performance for the metric.

	Model	Accuracy	Precision	Recall	F1
10-fold CV	SVC	**0.697 (0.095)**	**0.722 (0.091)**	0.660 (0.120)	0.678 (0.084)
	LR	0.632 (0.120)	0.645 (0.136)	0.656 (0.131)	0.647 (0.121)
	RF	0.668 (0.101)	0.705 (0.156)	**0.704 (0.114)**	**0.686 (0.084)**
Test Set	SVC	0.634	0.657	0.622	0.639
	LR	0.620	0.600	0.618	0.609
	RF	**0.746**	**0.771**	**0.730**	**0.750**

### Using GPT-3 embeddings

The classification performance of the GPT-3 embedding models is shown in Table 2 for the 10-fold CV (top, unshaded) and for the evaluation on the test set (bottom, shaded). Several observations can be made: (1) the use of GPT-3 embeddings yields a substantial improvement in performance when compared to the acoustic feature-based approach (Table 1); (2) the Babbage outperforms the Ada, a result consistent with the general notion that larger model is more powerful in various tasks [27]; (3) the performance of the 10-fold CV is comparable to that for the evaluation on the test set on the hold-out test set; and (4) direct comparison with the best baseline of the classification accuracy of 0.6479 [21] on the same test set reveals that GPT-3 performs remarkably well with the best accuracy of 0.8028 by SVC, showing clear advantage of using GPT-3 models.

**Table 2:** Model performance obtained by the 10-fold CV (top, unshaded) where the mean (standard deviation) are reported, and evaluated on test set (bottom, shaded) for AD classification using text embeddings from the GPT-3 base models (Babbage and Ada). Bold indicates the best overall performance of each metric separately for the top and the bottom panels.

	Embeddings	Model	Accuracy	Precision	Recall	F1
10-fold CV	Ada	SVC LR RF	0.788 (0.075) 0.796 (0.107) 0.734 (0.090)	0.798 (0.109) 0.798 (0.126) 0.738 (0.109)	0.819 (0.098) **0.835 (0.129)** 0.763 (0.149)	0.799 (0.066) 0.808 (0.100) 0.743 (0.103)
	Babbage	SVC LR RF	0.802 (0.054) **0.809 (0.112)** 0.760 (0.052)	0.823 (0.092) **0.843 (0.148)** 0.780 (0.102)	0.804 (0.103) 0.811 (0.091) 0.781 (0.110)	0.806 (0.053) **0.818 (0.091)** 0.770 (0.047)
Test Set	Ada	SVC	0.788	0.708	**0.971**	0.819
		LR	0.718	0.653	0.914	0.762
		RF	0.732	0.690	0.829	0.753
	Babbage	SVC	**0.803**	**0.723**	**0.971**	**0.829**
		LR RF	0.718 0.761	0.647 0.714	0.943 0.857	0.767 0.779

To examine how the GPT-3 based text embeddings fare with the fine-tuning approach, we use the GPT-3 Babbage as the pretrained model and fine tune it with the speech transcripts. The results are shown in Table 3 for both the 10-fold CV and evaluation on the test set. We see from Table 3 that, while the overall performance is comparable for both the 10-fold CV and the evaluation on the test set, the fine-tuned Babbage model underperforms the GPT-3 based text embeddings, a result in line with the recent findings that GPT-3 embedding model is even competitive with fine-tuned models [27]. We note, however, that there is no statistically significant difference of the accuracy between the finetuned model and the GPT-3 embedding based on a Kruskal-Wallis *H*-test (*H* = 0.8510, *p* > 0.05).

**Table 3:** Results for the fine-tuned GPT-3 Babbage model obtained by the 10-fold CV where the mean (standard deviation) are reported, and evaluated on test set for AD classification.

	Accuracy	Precision	Recall	F1
10-fold CV	0.797 (0.058)	0.810 (0.127)	0.809 (0.071)	0.797 (0.105)
Test Set	0.803	0.806	0.806	0.806

### Combination of acoustic features and GPT-3 embeddings

To evaluate whether the acoustic features and the text embeddings can provide complementary information to augment the AD classification, we combine the acoustic features from speech audio data and the GPT-3 based text embeddings by simply concatenating them. Table 4 shows the results for both the 10-fold CV and evaluation on the test set for different machine learning models. With additional acoustic features, we observe only marginal improvement in the classification performance on the 10-fold CV. There is no clear difference in predicting the test set in terms of accuracy and F1 score when the acoustic features are combined with GPT-3 based text embeddings, but we instead observe higher precision at the expense of lower recall. This observation indicates that the combined approach could be well-suited in screening AD when high precision is much more important than the recall.

**Table 4:** Model performance for the 10-fold CV with standard deviation and the evaluation on test set using a combination of the GPT-3 Babbage embeddings and the acoustic features.

	Model	Accuracy	Precision	Recall	F1
10-fold CV	SVC	**0.814 (0.115)**	**0.838 (0.133)**	0.802 (0.136)	**0.814 (0.119)**
	LR	0.800 (0.108)	0.831 (0.137)	**0.803 (0.097)**	0.809 (0.093)
	RF	0.731 (0.121)	0.741 (0.141)	0.762 (0.119)	0.745 (0.109)
Test Set	SVC	**0.802**	**0.971**	0.723	**0.829**
	LR	0.676	**0.971**	0.607	0.747
	RF	0.788	0.914	0.727	0.810

### Comparison of acoustic features with GPT-3 embeddings

**Fig 2:**
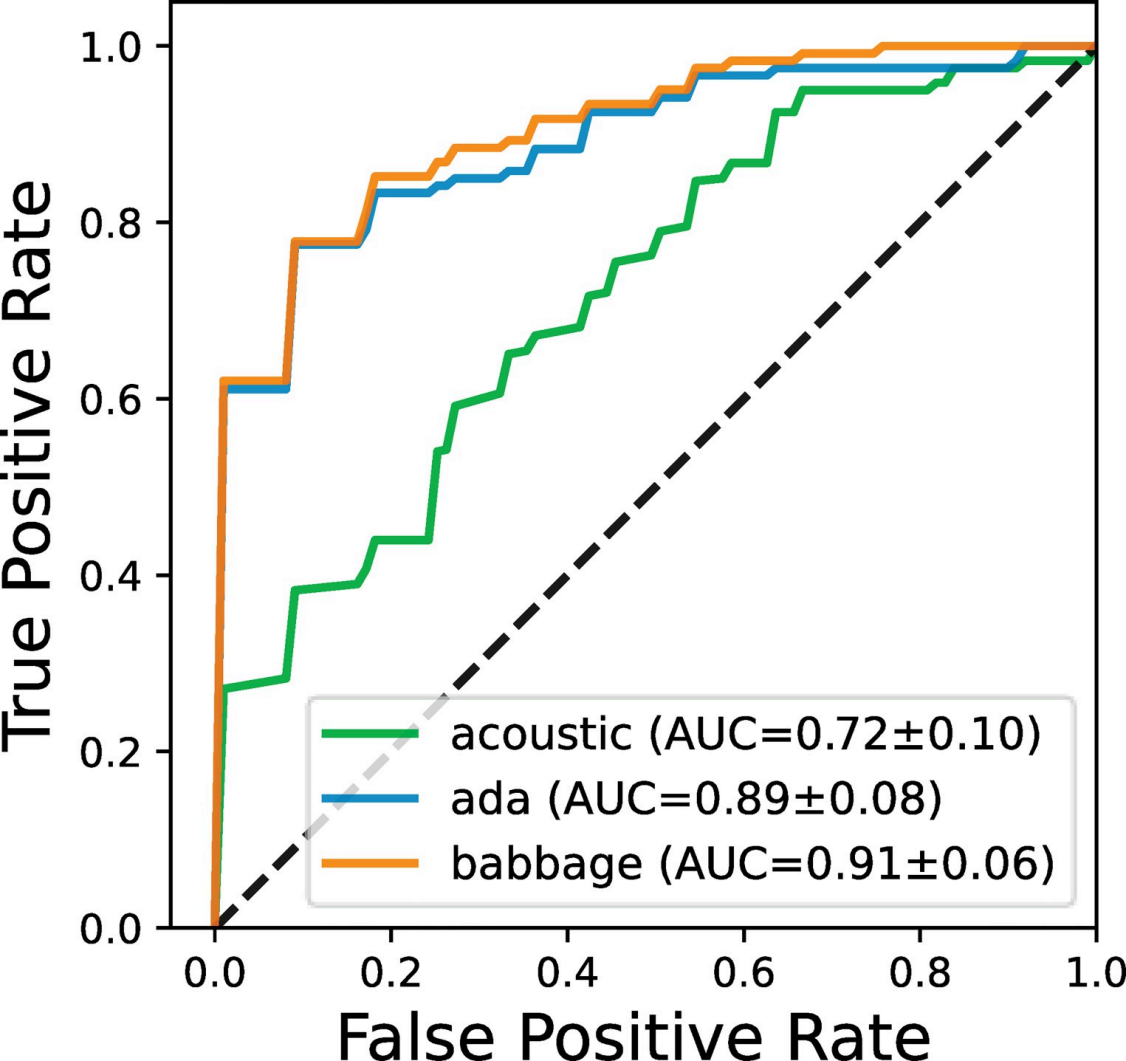
ROC curves, along with the averaged AUC scores and standard deviations, obtained by the 10-fold CV for the best acoustic, Ada and Babbage embedding models.

To compare the acoustic features with the GPT-3 embeddings, we perform further analysis based on the performance measurement of the area under the Receiver Operating Characteristic (ROC) curve (AUC). Fig 2 shows the ROC curves for RF model using the acoustic features (the best-performing acoustic model) and the GPT-3 embeddings (both Ada and Babbage). The mean and standard deviation of AUCs from the 10-fold CV are also reported, which indicate that the GPT-3 embeddings outperform the RF model using the acoustic features and the Babbage is marginally better than Ada. The Kruskal-Wallis *H*-test reveals a significant difference between the GPT-3 embeddings and the RF acoustic model (*H* = 5.622, *p* < 0.05).

### Comparison with several existing models

We benchmark our proposed GPT-3 embedding (Babbage) method against other state-of-the-art AD detection models. The existing methods include the studies from Luz et al [21], Balagopalan & Novikova [8] and Pan et al [31], which all used the ADReSSo Challenge data. The models selected are all trained based on the 10-fold CV and evaluated on the same unseen test set to ensure fair comparison. For example, we do not include Model 4 & 5 in Pan et al [31] as the models were trained by holding out 20% of the training set. Instead, we select the best model (Model 2), which was trained using 10-fold CV. The comparison is presented in Table 5, from which we can see that our method overall outperforms all other models in terms of accuracy, recall, and F1 score, though the precision is relatively low.

**Table 5:** Performance comparison between our model and other models on the ADReSSo 2021 unseen test set.

	Model	Accuracy	Precision	Recall	F1
GPT-3 Embedding (ours) Pan et al 2021 Balagopalan et al 2021 Luz et al 2021	SVC BERT_base_ SVC SVC	0.803 0.803 0.676 0.789	0.723 0.862 0.636 0.778	0.971 0.714 0.800 0.800	0.829 0.781 0.709 0.789

### MMSE Score Prediction

We perform the regression analysis using three different models: Support Vector Regression (SVR), Ridge Regression (Ridge) and Random Forest Regressor (RFR). The regression results, reported as root mean squared error (RMSE), using acoustic features and text embeddings from GPT-3 (Ada and Babbage) are shown in Tables 6 and 7, respectively. In each table, we provide the RMSE scores of the MMSE prediction for both the 10-fold CV and evaluation on the test set.

**Table 6:** MMSE prediction in terms of RMSE scores for three different models (SVR, Ridge and RFR) using acoustic features on the 10-fold CV (top) with standard deviation and on the inference on test set (bottom). Bold indicates the best RMSE score.

	Model	RMSE
10-fold CV	SVR	7.049 (2.355)
	Ridge	**6.768 (1.524)**
	RFR	6.901 (1.534)
Test Set	SVR	6.285
	Ridge	**6.250**
	RFR	6.434

**Table 7:** MMSE prediction in terms of RMSE scores for three different models (SVR, Ridge and RFR) using text embeddings from GPT-3 (Ada and Babbage) on the 10-fold CV (top) with standard deviation and on the inference on test set (bottom). Bold indicates the best RMSE score.

	Embeddings	Model	RMSE
10-fold CV	Ada	SVR	6.097 (2.057)
		Ridge	6.058 (1.298)
		RFR	6.300 (1.129)
	Babbage	SVR	5.976 (1.173)
		Ridge	**5.843 (1.037)**
		RFR	6.330 (1.032)
Test Set	Ada	SVR	5.6307
		Ridge	5.8735
		RFR	6.0010
	Babbage	SVR	5.4999
		Ridge	**5.4645**
		RFR	5.8142

With acoustic features, Table 6 shows that Ridge has the lowest RMSE score (6.2498) for MMSE prediction on the evaluation on the test set and the lowest RMSE of 6.7683 on the 10-fold CV. With the GPT-3 based text embeddings, Table 7 shows that Babbage has better prediction performance than Ada in terms of RMSE score in both the 10-fold CV and evaluation on the test set. When comparing the overall regression results in relation to what kinds of features are used, the GPT-3 based text embeddings provide clear advantage, as they always outperform the acoustic features.

## Discussion

The current NLP landscape has been revolutionized by large language models [23,26]. GPT- 3, a specific language model produced by OpenAI [26], is particularly powerful in encoding a wealth of semantic knowledge about the world and producing a high-quality vector representation (embedding) that lends itself well to discriminative tasks [27]. Given its impressive performance, we probe in this work the ability of GPT-3 to predict dementia from speech by utilizing the vast semantic knowledge encoded in the model. Our results demonstrate that the text embedding, generated by GPT-3, can be reliably used to not only detect individuals with AD from healthy controls but also infer the subject’s cognitive testing score, both solely based on speech data. We further show that text embedding outperforms the conventional acoustic feature-based approach and even performs competitively with fine-tuned models. These results, all together, suggest that GPT-3 based text embedding is a promising approach for AD assessment and has the potential to improve early diagnosis of dementia. We should note that our study performed model development and internal validation based mainly on the ADReSSo Challenge data; thus, further independent external validation is needed to confirm our findings.

There are four GPT-3 models available to the public via the OpenAI API, each having different number of embedding size and parameter: Ada (1024 dimensions, 300M), Babbage (2048 dimensions, 1.2B), Curie (4096 dimensions, 6B) and Davinci (12288 dimensions, 175B). These models have different capabilities and price points. Ada is the fastest and most affordable model but is the least capable, while Davinci is the most powerful but is most expensive among the models. As of this writing, we note the OpenAI paid-API is now more affordable. It is expected that GPT-3 will be eventually made free available to the community. When the decision is made as to which model should be used, the embedding size and the number of parameters are two important factors that ought to be taken into consideration. In general, larger models incur higher cost in terms of storage, memory, and computation time, which inevitably has direct impact on the model deployment in real-world applications such as AD diagnosis. Given the budget consideration and especially small data sample in the ADReSSo Challenge, we decided to go with the Ada and Babbage models in this application. Otherwise, there is a risk of overfitting when the data are not abundant, especially with the larger models (Curie and Davinci). Indeed, when we tested with the Curie and Davinci, we found the model overfitting by observing almost perfect recall and extremely low precision in AD classification task. We note that, while large sample sizes certainly help, we have taken precautious steps to test model generalizability with both the 10-fold CV and evaluation on the test set to guard against the problem of small sample size.

Fine-tuning has become the *de facto* standard to leverage large pretrained models to perform downstream tasks [24,25,32]. When we used the GPT-3 Babbage as the pretrained model and fine-tuned it with the speech transcripts, we however did not see the improvement in performance, as generally expected. While our results are in line with the recent findings that GPT-3 embedding model performs competitively with fine-tuned models [27], there is a possibility that the underperformance could be due to the insufficient data available in this task, as it is well known that the fine-tuning may predispose the pretrained model to overfitting due to the huge model and relatively small size of the domain-specific data. Such a possibility remains to be tested in the future when more data is available.

There is a huge potential to develop and translate a fully deployable AI-driven speech analysis for early diagnosis of dementia and direct tailored interventions to individual needs. Despite promising, major challenges lie with data quality (inconsistency and instability), data quantity (limited data), and diversity. For any models to work well, we need to have a very large, diverse and robust set of data. Leveraging AI with the growing development of large-scale, multi-modal data such as neuroimaging, speech and language, behavioral biomarkers, and patient information on electronic medical records, will help alleviate the data problem and allow for more accurate, efficient, and early diagnosis [33].

Our AI model could be deployed as a web application or even a voice-powered app used at the doctor’s office to aid clinicians in AD screening and early diagnosis. When applying AI and machine learning to predict dementia in clinical settings, there are however a number of potential problems. First, the bias should be considered in model development. It is mandated to have speech data from around the world, in many different languages, to guard against this problem, and to ensure the models work equitably for all patients, regardless of age, gender, ethnicity, nationality and other demographic criteria. It is preferred to develop ethical and legal systems for the implementation, validation and control of AI in clinical care [34]. Second, the privacy is a major concern in this nascent field, particularly speech data, which can be used to identify individuals. Third, there is a need to establish trust in AI, especially pertinent to the so-called ‘black box’ problem. This often arises in machine learning models where even the developers themselves can’t fully explain, particularly which information are used to make predictions. This can be problematic in clinical practice to explain how a diagnosis of dementia is ascertained and what can determine personalized treatments. Explainable AI aims to address the questions about the decision-making processes. Therefore, it is important to acknowledge that AI is not a replacement for human, but rather provides augmented decision making in driving efficient care and helping make accurate diagnoses. Before the AI-driven technologies enter mainstream use in aiding the diagnosis of dementia, it is essential to have rigorous validation from large-scale, well-designed representative studies through multidisciplinary collaboration between AI researchers and clinicians. This will ultimately allow AI to improve early diagnosis, which is crucial to improve quality of life for individuals with dementia.

## Materials and methods

### Dataset description

The dataset used in this study is derived from the ADReSSo Challenge [21], which consists of set of speech recordings of picture descriptions produced by cognitively normal subjects and patients with an AD diagnosis, who were asked to describe the Cookie Theft picture from the Boston Diagnostic Aphasia Examination [6,35]. There are totally 237 speech recordings, with 70/30 split balanced for demographics, resulting in 166 and 71 in the training set and the test set, respectively. In the training set, there are 87 samples from AD subjects and 79 from non-AD (or healthy control) subjects. The datasets were matched so as to avoid potential biases often overlooked in assessment of AD detection methods, including incidences of repetitive speech from the same individual, variations in speech quality, and imbalanced distribution of gender and age. The detailed procedures to match the data demographically according to propensity scores were described in Luz et al. [21]. In the final dataset, all standardized mean differences for the age and gender covariates are < 0.001.

### Ethics statement

The studies involving human participants were reviewed and approved by DementiaBank consortium. All enrolled participants provided informed written consent to participate in this study. All data analyses in this work are conducted using the de-identified data.

### Computational approaches

At the core of our proposed approach (Fig 1B) is the text embedding from GPT-3 [26], which can be readily accessed via OpenAI Application Programming Interface (API). The OpenAI API, powered by a family of models with different capabilities and price points, can be applied to virtually any task that involves understanding or generating natural language or code. We use the GPT-3 for text embedding, which is powerful representation of the semantic meaning of a piece of text. We benchmark our GPT-3 embedding approach against both the conventional acoustic feature-based approach (Fig 1A) and the prevailing fine-tuned model.

### Text embeddings from GPT-3

Central to our approach is the innovative use of text embeddings, powered by GPT-3. To our knowledge, this is the first application of GPT-3 to predicting dementia from speech. In our approach (Fig 1B), we first convert voice to text using Wav2Vec 2.0 pretrained model [36], a state-of-the-art model for automatic speech recognition. We use the base model *wav2vec2-base-960h* that was pretrained and fine-tuned on 960 hours of Librispeech on 16 kHz sampled speech audio, which can be accessed from Huggingface [37]. Each audio file is loaded as a waveform with librosa [38], a python package dedicated to analyzing sounds. The waveform is then tokenized using *Wav2Vec2Tokenizer* and if necessary, divided them into smaller chunks (with the maximum size of 100,000 in our case) to fit into memory, which is subsequently fed into the *Wav2Vec2ForCTC* (a wav2vec model for speech recognition) and decoded as text transcripts.

GPT-3 based text embeddings are afterwards derived from the transcribed text obtained via *wav2vec2*. We use the endpoint in the OpenAI API, which is available to the registered researchers, to access GPT-3 embedding models [27]. These embeddings entail meaningful vector representations that can capture lexical, syntactic, and semantic properties useful for dementia classification. It is recently shown that with GPT-3 based text embeddings new state-of-the-art results can be achieved in a variety of tasks including semantic search, clustering, and classification [27].

There are four GPT-3 models on a spectrum of embedding size: Ada (1024 dimensions), Babbage (2048 dimensions), Curie (4096 dimensions) and Davinci (12288 dimensions). Davinci is the most powerful but is more expensive than the other models, whereas Ada is the least capable but is significantly faster and cheaper. As of this writing, the embeddings are billed at 10 times of the base prices. Specifically, they charge $0.2 and $0.004 per 1000 tokens for the largest model (Davinci) and the smallest model (Ada), respectively. These embeddings are finally used as features to train machine learning models for AD assessment. Given the cost consideration, especially the small sample size in the ADReSSo Challenge, we report the results obtained by the Ada and Babbage models.

### Acoustic feature extraction from speech

Conventional acoustic feature-based approach (Fig 1A) will be used as benchmark for comparison. The acoustic features considered are mainly related to temporal analysis (e.g. pause rate, phonation rate, periodicity of speech, etc.), frequency analysis (e.g. mean, variance, kurtosis of Mel frequency cepstral coefficients) and different aspects of speech production (e.g. prosody, articulation, or vocal quality). In this work, acoustic features are extracted directly from speech using OpenSMILE (open-source Speech and Music Interpretation by Large-space Extraction), a widely used open-source toolkit for audio feature extraction and classification of speech and music signals [39]. We primarily used the extended Geneva Minimalistic Acoustic Parameter Set (eGeMAPS) features due to their potential to detect physiological changes in voice production, as well as theoretical significance and proven usefulness in previous studies [40]. There are, in total, 88 features: the arithmetic mean and coefficient of variation of 18 low-level descriptors (e.g., pitch, jitter, formant 1–3 frequency and relative energy, shimmer, loudness, alpha ratio and Hammarberg index etc), 8 functionals applied to pitch and loudness, 4 statistics over the unvoiced segments, 6 temporal features, and 26 additional cepstral parameters and dynamic parameters. This feature set once obtained can be used directly as inputs to a machine learning model.

### Fine-tuning with speech transcripts

Fine-tuning is the prevalent paradigm for using LLMs [23–25] to perform downstream tasks. In this approach, the pretrained models such as the BERT (Bidirectional Encoder Representations from Transformers) [32], either some or all the model parameters, can be finetuned or updated with downstream task-specific data. Recent work has shown encouraging results with fine-tuned BERT for AD detection [20,41]. In this study, we will also benchmark our proposed GPT-based embedding approach against the mainstream use of fine-tuned model. As such, we use the GPT-3 as the pretrained model and fine-tune it with speech transcripts obtained by wav2vec2 from raw audio files.

To fine tune our own custom GPT-3 models, we use the OpenAI command-line interface, which is released to the public. We simply follow the instructions about fine-tuning, provided by OpenAI, to prepare the training data that consists of 166 paragraphs, totaling 19,123 words that are used to fine tune one of the base models (Babbage and Ada in our case) with speech transcripts. Tokens used to train a model are relatively cheaper, as billed at 50% of the base prices.

### Experimental tasks

#### AD vs non-AD classification

The AD classification task consists of creating a binary classification model to distinguish between AD and non-AD speech. The model may use acoustic features from speech, linguistic features (embeddings) from transcribed speech, or both. As such, we use (1) the acoustic features extracted from speech audio data, (2) the text embeddings from each GPT-3 base model (Babbage or Ada), and (3) the combination of both as inputs for three different kinds of commonly used machine learning models, including Support Vector Classifier (SVC), Random Forest (RF), and Logistic Regression (LR). We use the scikit-learn library for the implementation of these models [42]. The hyperparameters for each model are tuned using the 10-fold cross-validation. Specifically, there are two key parameters (the regularization parameter and the kernel coefficient) for SVC trained with a radial basis function kernel, the *L*2-penalty parameter for LR and two key parameters (the number of estimators and the maximum depth of the tree) for RF. As a comparison, we also fine tune the GPT-3 model (Babbage) with the speech transcripts to assess if the GPT-3 based text embeddings can be better used to predict the dementia.

#### MMSE score prediction

MMSE is perhaps the most common measure for assessing the severity of AD. We perform regression analysis using both the acoustic features and text embeddings from GPT-3 (Ada and Babbage) to predict the MMSE score. The scores normally range from 0 to 30, with scores of 26 or higher being considered normal [3]. A score of 20 to 24 suggests mild dementia, 13 to 20 suggests moderate dementia, and less than 12 indicates severe dementia. As such, the prediction is clipped to a range between 0 and 30. Three kinds of regression models are employed, including Support Vector Regression (SVR), Ridge regression (Ridge) and Random Forest Regressor (RFR). The models are similarly implemented with the scikit-learn library [42], with the hyperparameters for each model determined using grid-search 10-fold cross-validation on the training dataset.

### Performance evaluation

For AD classification task, the performance is evaluated by a panel of metrics such as the accuracy, precision, recall and F1-score, where the threshold of 0.5 is used. The ADReSSo Challenge dataset was already split into the training set and the test set, with 70% of samples allocated to the former and 30% allocated to the latter. To evaluate the generalization ability of the model, we have two ways to report the performance: 10-fold cross-validation (CV) and evaluation on the test set. The model is well calibrated before testing. The first way is to test for generalizability within a dataset using the 10-fold CV approach. This way, we partition all the available data (i.e., the entire data including the training set and test set) into three sets (training, validation and test sets) in an 80/10/10 ratio using the 10-fold CV. That is, we use 8-fold for training, 1-fold for validation, and the remaining for testing in each run. We report the average of the ten independent runs in which the test data is different in each run. As such, we can reduce the potential sampling bias where the results can depend on a particular random choice of the data sets. We also report the averaged AUC scores, along with the corresponding standard deviations over the 10-fold CV when comparing the different models using acoustic features, GPT-3 embeddings (both Ada and Babbage) for AD classification.

The second way to report the performance is that the model is evaluated on an unseen test set not used in any way during model development. Since we have a separate test set that was already set aside, we use it as the independent, held-out dataset. We still perform the 10-fold cross-validation, but only the existing training set. That is, we split the training dataset into ten folds, with 9 folds for training, and the remaining for validation to tune hyperparameters in each run for ten independent runs. We then fit the model on all the training dataset using the hyperparameters of the best model, and then use the final model on the held-out test data. The use of the held-out test data allows us to directly compare the different models as well as with Challenge baseline on the same dataset. We stress that the test dataset is different between 10-fold CV and evaluation on the test set.

For AD regression task, we similarly conduct the 10-fold CV and inference on the test set. We report root mean squared error (RMSE) for the MMSE score predictions on the testing data using the models obtained by 10-fold CV. The hyperparameters for each model are determined based on performance in grid-search 10-fold cross-validation on the training dataset.

In finetuning GPT-3 for the AD classification task, the hyperparameters we used are consistent with the recommendations by OpenAI for its Babbage model. Specifically, the hyperparameters available to be tuned include the number of epochs, batch size and learning rate multiplier. We vary the number of epochs from 1 to 5, learning rate multiplier between 0.02 to 0.2 and the batch size between 4 to 10 and compare with the results from default internal parameters originally set by OpenAI. It turned out that the recommended hyperparameters by OpenAI work best for the finetuning.

In doing the 10-fold CV, all the results we reported are the average of the ten folds, together with its standard deviation. The statistical significance between the models is performed via the Kruskal-Wallis *H*-test. We use the Kruskal-Wallis *H* test for sample comparison because it is non-parametric and hence does not assume that the samples are normally distributed.
